# Deep sequencing of HetR-bound DNA reveals novel HetR targets in *Anabaena* sp. strain PCC7120

**DOI:** 10.1186/s12866-014-0255-x

**Published:** 2014-10-03

**Authors:** Britt L Flaherty, David BF Johnson, James W Golden

**Affiliations:** Division of Biological Sciences, University of California San Diego, La Jolla, CA USA; Present address: Illumina, Inc., San Diego, CA USA; Present address: Peterson, Wilmarth, and Robertson, LLP, Washington, DC USA

**Keywords:** Cyanobacteria, Heterocyst development, HetR, ChIP-seq

## Abstract

**Background:**

*Anabaena* (also *Nostoc*) sp. strain PCC7120, hereafter *Anabaena*, is a cyanobacterium that fixes atmospheric N_2_ in specialized cells called heterocysts. Heterocyst differentiation is regulated by a homodimeric transcription factor, HetR. HetR is expressed at a basal level in all cells but its expression increases in differentiating cells early after nitrogen deprivation. HetR is required for heterocyst development, and therefore nitrogen fixation and diazotrophic growth. Overexpression of HetR leads to multiple contiguous heterocysts (Mch phenotype). HetR binds *in vitro* to DNA fragments upstream of several genes upregulated in heterocysts, including *hetZ*, *hetP*, *hepA*, *patS*, *pknE*, and *hetR* itself. HetR binds an inverted repeat sequence upstream of a few of these genes; however, HetR binds to promoters that do not contain this sequence, such as the promoter regions for *patS* and *pknE*.

**Results:**

We employed chromatin pull-down and deep sequencing (ChIP-seq) to globally identify HetR DNA targets *in vivo* at six hours after fixed-nitrogen deprivation. We identified novel DNA binding targets of tagged HetR-6xHis and defined a consensus HetR binding site from these HetR target sequences. Promoter-*gfp* reporter fusions were used to determine the spatiotemporal expression of four potential HetR-target genes. The promoter region for asr1469 was expressed transiently in differentiating heterocysts, alr3758 was upregulated in heterocysts, asl2028 was expressed in vegetative cells, and alr2242 was derepressed in vegetative cells of a *hetR* mutant strain.

**Conclusions:**

In addition to identifying known HetR target genes *hetR* and *hetP*, the ChIP-seq data were used to identify new potential HetR targets and to define a consensus HetR-binding site. The *in vivo* ChIP-seq analysis of HetR’s regulon suggests a possible role for HetR in vegetative cells in addition to its role in heterocyst development. The potential HetR target genes identified in this study provide new subjects for future work on the role of HetR in gene regulation.

**Electronic supplementary material:**

The online version of this article (doi:10.1186/s12866-014-0255-x) contains supplementary material, which is available to authorized users.

## Background

*Anabaena* and *Nostoc* species fix atmospheric N_2_ into ammonia in specialized nitrogen-fixing cells called heterocysts at spaced intervals along filaments of photosynthetic vegetative cells. In response to nitrogen deprivation, a transcriptional cascade controls the differentiation of heterocysts along the filament. HetR is a key transcription factor required for heterocyst development and diazotrophic growth. HetR mutant strains are blocked at an early stage of heterocyst differentiation [1-3]. In *Anabaena* sp. strain PCC7120, overexpression of HetR results in multiple contiguous heterocysts (Mch phenotype) in nitrate-containing medium, showing that HetR alone can trigger heterocyst development and override normal nutritional queues [4]. HetR is expressed at a basal level in vegetative cells and its expression increases in heterocysts early after nitrogen deprivation [3-5]. The crystal structure of HetR from *Fischerella* strain MV11 shows that HetR forms a dimer and contains helix-turn-helix motifs in the N-terminal regions of the monomers, forming a DNA-binding region flanked by globular histidine-rich flaps [6,7]. The C-terminal regions form a hood associated with the central core.

HetR has been shown to bind DNA with electrophoretic mobility shift assays, but its DNA target sequence is still not fully understood [7-11]. HetR binds *in vitro* to DNA fragments upstream of several genes upregulated in heterocysts, including *hetZ*, *hetP*, *hepA*, *pknE*, *patS*, and *hetR* itself [8-11]. HetR binds strongly to a 17-bp inverted repeat, 5′-GAGGGGTCTAACCCCTC-3′, in the *hetP* promoter, but this sequence is not found in other HetR-regulated promoters [9]. A consensus target sequence derived from *hetP* promoters from several cyanobacterial strains was determined to be 5′-tnantngnGGGtcaanCCCanca-3′, and crystal structures of HetR in a complex with three different lengths of a DNA target based on this consensus sequence revealed details of the protein-DNA interaction that define a requirement for the sequence GGGnnnnnCCC, where n can be any base [7]. A study that identified the HetR binding site upstream of *hetZ* suggested that the HetR binding site is related to the imperfect palindrome GGGTCTAgCCCagCA [10], but this site is not upstream of all known HetR targets, including the *patS* gene, which is involved in heterocyst pattern regulation [12-14].

A genome-wide map of transcription start sites (TSS) in the wild type and a *hetR* mutant strain after nitrogen deprivation for 8 h identified a “DIF” (differentiation related) TSS category that depends on HetR for upregulation [15]. A DIF^+^ sequence motif, TCCGGA, centered near the −35 position, was found in many heterocyst-specific promoters, but the previously identified HetR binding site was not found to be conserved in these promoters [15]. This study could not distinguish between direct and indirect effects on expression levels.

Genes encoding HetR are conserved in conjunction with *patS* genes in both heterocystous and nonheterocystous cyanobacteria [16]. PatS and HetR are thought to act together to regulate the spacing of heterocysts [8,17,18]. A small pentapeptide RGSGR motif in PatS and HetN is sufficient to inhibit heterocyst formation [12-14,19] and PatS peptides can bind directly to the HetR dimer with PatS-6 binding the tightest [17,20]. Because HetR and PatS are conserved in cyanobacteria that do not form heterocysts, and because HetR is expressed at low levels in vegetative cells, it is possible that HetR and PatS serve a function in addition to their known roles in heterocyst development [16]. HetR has two protein interaction domains, suggesting that other proteins may modulate HetR’s activity and DNA binding [6]. However, very little is known about HetR’s possible role outside heterocyst development or about its target genes in nonheterocystous cyanobacteria.

We employed chromatin pull-down (ChIP, originally for chromatin immunoprecipitation) and deep sequencing to give a global view of HetR’s *in vivo* DNA-binding sites in both vegetative cells and proheterocysts six hours after induction. ChIP assays have been used to map DNA binding sites for RNA polymerase and two transcription factors in the cyanobacterium *Synechococcus elongatus* PCC7942 [21,22]. A recent study used ChIP-seq to examine the NtcA DNA binding sites in *Anabaena* sp. PCC7120 at three hours after withdrawal of combined nitrogen [23]. This study found NtcA binding regions associated with over two thousand genes. We applied ChIP-seq to HetR because identifying its potential target sites and its regulon would be a significant step forward in understanding the set of genes required for heterocyst development. We isolated 6xHis-tagged HetR bound to DNA at six hours after the removal of combined nitrogen and used deep sequencing to identify all regions of the genome enriched for HetR binding. ChIP-seq, which identifies a protein’s *in vivo* binding sites under specific growth conditions, has the potential of identifying HetR targets that would not be identified with other methods. We developed a ChIP protocol for HetR in *Anabaena* and optimized the downstream data analysis. We used ChIP-seq to identify potential HetR target genes and to produce a consensus DNA-binding site, and we used promoter-*gfp* fusions to study the spatial and temporal expression of four of these genes.

## Results and discussion

To identify potential members of the HetR regulon at an early stage of heterocyst development and to determine a consensus HetR binding site, we identified HetR DNA targets at six hours after combined-nitrogen deprivation with ChIP-seq. HetR is known to be expressed and regulate “early” target genes at this time point [10,11,24]. Cells from a wild-type (WT) control strain and *hetR* mutant strain UHM103 carrying pAM4375, which expresses tagged HetR-6xHis and produced a nearly normal heterocyst phenotype, were exposed to crosslinking agents *in vivo* and His-tagged HetR bound to its DNA targets was then affinity precipitated. ChIP DNA samples were then subjected to deep sequencing on the Illumina HiSeq platform, and reads were aligned to the *Anabaena* PCC7120 genome. The CLC Genomics Workbench 5 ChIP-seq algorithm was used to search for ChIP peaks with the WT sample as a nonspecific control. The algorithm found 38 ChIP peaks with a 5% false discovery rate (FDR) and a 100-bp window for the Poisson distribution (Additional file 1: Table S1).

### HetR binding site consensus sequence

From the 38 identified ChIP peaks, we analyzed each peak region by eye to confirm a 3-fold enrichment in reads of the ChIP peak over wild type and a twin peak morphology, indicative of a transcription factor binding to DNA. In addition, we excluded peaks that were not upstream of a gene that is misregulated in a ∆*hetR* background, as determined by RNA-seq analysis of a ∆*hetR* mutant at 0 and 6 hours after nitrogen deprivation (unpublished data), and peaks that were not in a 5′ untranslated region (UTR) or near a potential TSS as identified by our RNA-seq data [25] or by differential RNA-seq [26]. We queried the resulting 26 ChIP regions (Table 1) with FiMO (Find Individual Motif Occurrences) for the previously defined HetR binding site [10,27] with a p value of 10^−4^. FiMO found 59 potential HetR binding sites near 20 of the 26 ChIP peaks (Additional file 2: Table S2). Several ChIP peaks, for example the broad peaks associated with the *hetR* and *hetP* genes, contained multiple potential HetR binding sites in addition to the previously characterized sites [8-10]. HetR dimers might individually bind to these multiple sites, but it is possible that multiple binding sites could result in HetR tetramer formation [7], which could be involved in the regulation of certain promoters.

**Table 1:** **HetR ChIP-seq peak regions** ^**a**^

**Chromosomal coordinates**	**Length base pairs**	**Gene to left**	**Fold change expression in ∆** ***hetR*** **6 h**	**Gene to right**	**Fold change expression in ∆** ***hetR*** **6 h**	**HetR binding site**
0136127..0136558	431	all0131	Low coverage	alr0132	−1.1	Yes
0613436..0613919	483	*patA* (all0521)	−8.0	alr0522^d^	−5.6	Yes
1729122..1729569	447	all1467	1.4	alr1468	Low coverage	Yes
1732738..1733693	955	asr1469^c^	1.7	alr1470	−3.1	Yes
1734763..1735224	461	all1471	−2.3	all1472^c^	−1.6	Yes
1788499..1788932	433	alr1527	−5.8	alr1528^d^	NA^b^	Yes
2427714..2428147	433	all2027	3.8	asl2028	−1.5	Yes
2691579..2692024	445	all2239	−4.0	alr2240	−7.1	Yes
2820376..2822006	678	alr2338^c^	−4.4	*hetR* (alr2339)	−131	Yes
3431297..3432038	741	*hetC* (alr2817)^c^	NA	*hetP* (alr2818)	−4.4	Yes
3627499..3627977	478	alr2986^c^	−3.0	alr2987^d^	NA	Yes
3695599..3696450	851	asr3053^c^	2.1	all3054^d^	NA	Yes
3821175..3821606	431	alr3156^c^	NA	*aphA* (alr3157)	−7.7	Yes
4383426..4383866	440	*avaIR* (all3631)^d^	−1.3	*avaIM* (all3632)^c^	−1.9	Yes
4539927..4540364	437	alr3757^c^	NA	alr3758	11 / 0	Yes
4740704..4741151	447	alr3926^c^	NA	all3927^c^	−2.9	Yes
5064797..5065242	445	asr4228^c^	NA	alr4229	−7.5	Yes
5303024..5303480	456	all4424	5.2	all4425^c^	1.7	Yes
5309952..5310383	431	all4430	4.9	all4431^c^	10.3	Yes
5355615..5356047	432	alr4469^c^	−1.3	asr4470	−10.8	Yes
0374646..0375090	444	all0326	Low coverage	all0327^c^	NA	No
2274728..2275590	862	alr1903^c^	−4.9	cyaB2 (all1904)^c^	NA	No
2693489..2693944	455	alr2241^c^	NA	alr2242	−13.4	No
2706756..2707187	431	*gvpA* (asl2254)	−6.3	asr2255	Low coverage	No
4857231..4857672	441	alr4031^c^	NA	alr4032	Low coverage	No
6129044..6129475	431	all5131	−4.2	all5132^c^	−2.5	No

^a^See text for details on peak selection criteria. If no footnote for gene, then ChIP peak region is within 500 bp upstream of ORF.

^b^NA, not applicable because ChIP peak region is unlikely to regulate ORF expression.

^c^ChIP peak region is downstream of potential TSS.

^d^ChIP peak region is >500 bp upstream of ORF.

**Figure 1:**
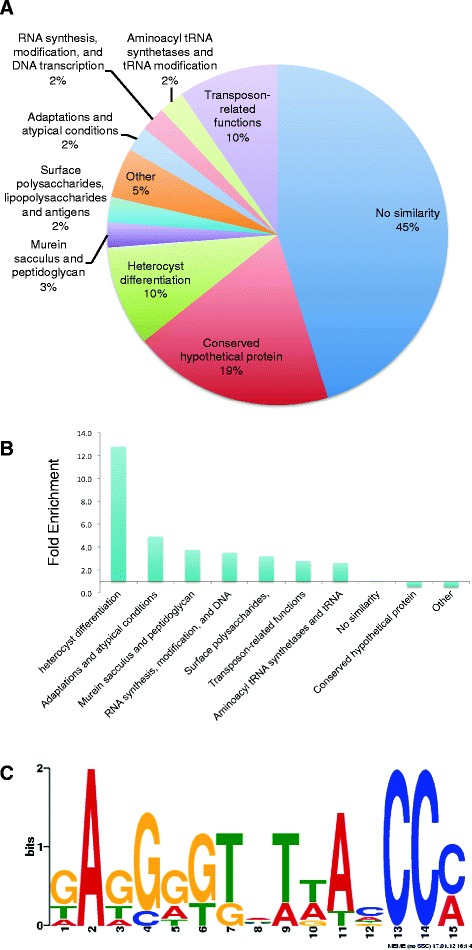
**Gene ontology categories for HetR ChIP peaks, and the consensus HetR binding site. A**. GO Term gene categories for HetR target genes identified by ChIP. Significant enrichment in the GO Term categories “Heterocyst Differentiation” and “Transposon Related Functions” was seen in potential targets of HetR as identified by ChIP. These two categories were enriched at least 3-fold over their relative proportion in the genome as a whole. Each GO term is labeled with the percent of ChIP hits that fall within that category. **B**. Fold enrichment of HetR-ChIP target Go Terms over their representation in the *Anabaena* genome. For example, the category “heterocyst differentiation” is represented over 12 times more often in the HetR-ChIP targets than would be expected from a random sampling of the genome. **C**. HetR consensus binding site in MEME format. Twenty ChIP peaks were queried with FiMO for the previously identified HetR binding site GGGTCTAgCCCagCA. 59 discovered sites were then aligned with the MEME suite to produce a consensus HetR binding site.

We used the MEME suite to align the 59 discovered potential HetR binding sites identified with FiMO to produce a consensus HetR binding site (Figure 1C). A key difference between the ChIP-seq consensus HetR binding site and previously identified sites [10] include a conserved “A” residue two bases upstream of the triple G repeat. The ChIP-seq consensus HetR binding site shows little to no conservation of the nucleotides downstream of the CCC bases.

We performed mobility shift assays with affinity purified GST-HetR protein [11] and biotin-labeled oligonucleotides for nine potential HetR binding sites. Although we obtained a shifted band for a *hetP* positive control fragment, we failed to obtain distinct shifted bands for the newly identified potential targets (data not shown). However, for six of the nine oligonucleotides, there was a clear decrease in the free probe band and a shifted more slowly migrating diffuse smear in the presence of GST-HetR compared to a GST control, which indicates interaction between these DNA probes and the HetR protein. These six binding sites were upstream of the genes *patA*, all1467/alr1468, asr1469, all2240, alr2242, and all5131. Alternative biophysical methods will be required to further characterize the HetR interaction with these DNA binding sites.

The 26 ChIP peak regions were near some genes that have been reported to contain HetR binding sites, including *hetR*, *hetP*, and *patA*, but most of the genes represent potential new HetR targets (Table 1). A few genes that have been shown to be regulated by HetR, including *hetZ*, *patS*, and *pknE*, were not associated with the ChIP-seq regions identified in this study. However, HetR is thought to potentially interact with other factors [6], and these partners may affect binding and could be different at different times and in different cell types. Because our ChIP sample was collected at 6 h after nitrogen removal, the consensus binding site we have defined would be dependent on HetR concentration and other factors present at that time point. Furthermore, our ChIP data are from HetR present in all cells in each filament, which includes both early proheterocysts and vegetative cells. Therefore, our binding sites may be skewed towards HetR targets in vegetative cells compared to previous analyses, which focused on HetR’s role in regulating heterocyst-specific genes.

### GO term enrichment in HetR targets

Genes in seven Gene Ontology (GO Term) categories were enriched in our ChIP peak sample set with respect to their abundance in the genome, such as “heterocyst differentiation,” “adaptations and atypical conditions,” “transcription,” and “transposon related functions” (Figure 1). As expected, genes involved in heterocyst differentiation, including genes such as *hetR*, *hetP*, *hetC*, and *patA*, were enriched as targets for HetR. We did not initially expect transposases to be potentially regulated by HetR. However, genes for transposon-related functions were enriched in our HetR-ChIP data and were also upregulated in response to nitrogen deprivation in previously published RNA-seq data [25]. Seven of the original 38 HetR ChIP peaks were associated with transposase genes. It seems unlikely that these interactions are part of the regulatory network that regulates the response to nitrogen deprivation or heterocyst development, and it is probably more likely that these transposases have hijacked HetR regulation as a signal of cellular stress, which is known to activate transposons in other systems.

### Promoter-*gfp* fusions for HetR targets

**Figure 2:**
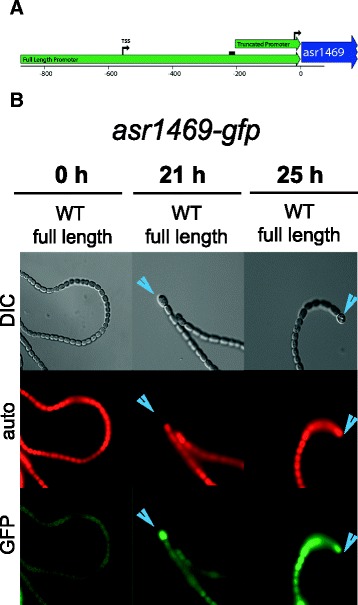
**asr1469 promoter-** ***gfp*** **was transiently upregulated in heterocysts. A**. Map of full length and truncated promoter regions upstream of asr1469 that was used to drive GFP reporter expression. Bent arrow indicates a putative transcription start site from RNA-seq data [25], bent arrow labeled TSS indicates start site identified by differential RNA-seq [26], black bar indicates HetR binding site. **B**. GFP fusions show transient expression of the full-length asr1469*-gfp* reporter fusion in heterocysts. *Anabaena* WT cells were imaged at 0, 21, and 25 hours after nitrogen deprivation in DIC (top), TRITC/autofluorescence (middle), and GFP (bottom) channels. At 21 hours after nitrogen deprivation, 63% of heterocysts showed an increase in fluorescence compared to vegetative cells. By 25 hours after nitrogen deprivation, the majority of heterocysts showed decreased or no GFP fluorescence. Heterocysts are marked with a blue arrowhead.

**Figure 3:**
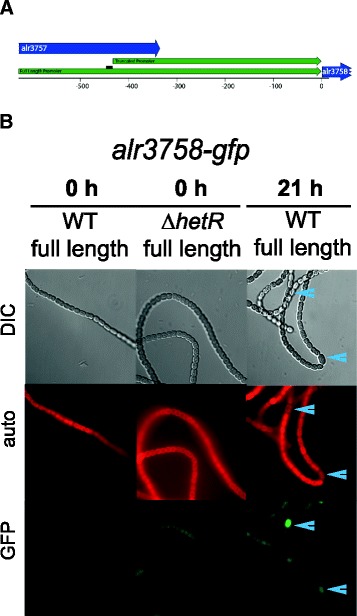
**alr3758 promoter-** ***gfp*** **was upregulated in heterocysts. A**. Map of full length and truncated promoter regions upstream of alr3758 that were used to drive GFP reporter expression. Black bar indicates HetR binding site. **B**. Images show *Anabaena* cells carrying the reporter construct at 0 h for both the WT and ∆*hetR* mutant, and at 21 h for the WT after nitrogen deprivation in the DIC (top), TRITC/autofluorescence (middle), and GFP (bottom) channels. There was very low to no expression in the WT and ∆*hetR* backgrounds at 0 h. In the WT background at 21 hours after nitrogen deprivation, heterocysts showed an increase in fluorescence. Heterocysts are marked with a blue arrowhead.

**Figure 4:**
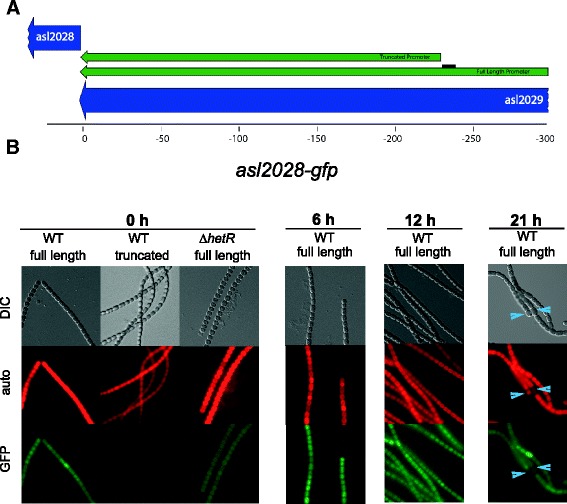
**asl2028 promoter-** ***gfp*** **was expressed in vegetative cells. A**. Map of full length and truncated promoter regions upstream of asl2028 that were used to drive GFP reporter expression. Black bar indicates HetR binding site. **B**. The asl2028 full length promoter fused to *gfp* in WT cells was expressed in vegetative cells (0 h) and showed a slight increase in expression in vegetative cells at 6, 12, and 21 hours after nitrogen deprivation. The asl2028-*gfp* reporter was not expressed in heterocysts at 21 hours after nitrogen deprivation. The full-length asl2028 promoter was expressed at a lower level in the ∆*hetR* mutant background compared to its expression in WT cells.

We chose four potential HetR targets for further analysis of their temporal and spatial regulation in response to nitrogen deprivation, and also discuss *patA*, which was identified in our ChIP-seq experiments (Table 2). Four of these targets, *patA*, asr1469, asl2028, and alr3758 contain a potential HetR-binding sequence while all2242 does not. Each of these genes was downregulated at either 0 or 6 h in a ∆*hetR* strain, and alr2242 and alr3758 are upregulated more than 2-fold in response to nitrogen deprivation (Table 2). To analyze the temporal and spatial expression of these genes in response to nitrogen deprivation, we fused the potential promoter region of each gene to a *gfp* reporter gene in both wild-type *Anabaena* and strain UHM103, a markerless knockout mutant of *hetR* [28]. For the three genes that have putative HetR binding sites, asr1469, asl2028, and alr3758, we also fused a truncated version of the promoter, missing the HetR binding site, to *gfp*. For each of the truncated promoter fusions (maps shown in Figures 2A, 3A, and 4A), we detected little to no GFP fluorescence (data not shown). It is possible that by truncating these promoters, we also removed other signals in addition to the HetR binding site that are important for gene expression.

**Table 2:** **Four potential HetR targets chosen for further analysis and** ***patA***

**Region location**	**RNA-seq RPKM fold change** ^**a**^					**Gene function**	**Potential HetR binding site**
	**WT 0 h/∆** ***hetR*** **0 h**	**WT 6 h/∆** ***hetR*** **6 h**	**WT 0 h/WT 6 h**	**WT 0 h/WT 12 h**	**WT 0 h/WT 21 h**		
*patA* (all0521)	−13	−8.0	−1.8	1.6	1.4	Pattern formation	TTGGCTCAAACCCAT
asr1469	−4.0^b^	1.7^b^	--Low coverage--			Small peptide	TTGGGTCTAACTTAT
asl2028	−2.5	−1.5	−3.7	1.2	1.4	Unknown	TCGGGGTAACAATCC
alr2242	1.3	−13.4	1.5	1.1	2.7	Possible NTPase	None found
alr3758	Low coverage	11 / 0^c^	12	3.9	7.7	Anti sigma factor antagonist	TTGGGTAAACCTGCT

^a^WT RPKM data from Flaherty et al. [25].

^b^Low coverage.

^c^11 reads in WT at 6 h dropped to 0 in the ∆*hetR* mutant.

The asr1469 gene was of interest because it is near three HetR ChIP-seq peaks and is present on the *fdxN* element, which is excised from the chromosome in heterocysts [24,29]. The asr1469 gene encodes a small protein conserved in a few diverse cyanobacteria but with no known domain homologies. Genome context provides no additional information because unknown and hypothetical genes surround asr1469. The full-length asr1469 promoter driving *gfp* in WT cells produced weak GFP fluorescence in all cells in N + conditions (Figure 2). After nitrogen deprivation, GFP fluorescence was increased in 63% of heterocysts at 21 hours, while the remaining heterocysts showed either GFP fluorescence similar to vegetative cell levels (27%) or no GFP signal (10%). However, by 25 hours after the removal of combined nitrogen, only 25% of heterocysts showed high GFP fluorescence and 65% showed no GFP fluorescence, while the remaining 10% of heterocysts showed GFP fluorescence similar to that of vegetative cells. Therefore, the asr1469 gene appears to be transiently upregulated in heterocysts during heterocyst differentiation while maintaining a lower level of expression in vegetative cells. Very low to no GFP expression from the full-length asr1469 promoter was observed in a ∆*hetR* background (not shown).

RNA-seq data showed that asr1469 is expressed at a low level with a four-fold decrease in expression in the ∆*hetR* strain at 0 h, but no significant regulation of the gene in response to nitrogen deprivation. It is likely that the changes in transcription of asr1469 seen with the *gfp*-reporter fusion are too transient to be detected with RNA-seq of RNA from whole filaments measured at discreet time points. However, the promoter-*gfp* fusion suggests that asr1469 transcription is dependent on HetR because expression of GFP required both HetR protein and the full-length promoter containing the putative HetR binding site (not shown). Because the function of asr1469 is unknown, the reason for its transient up-regulation in heterocysts is unclear.

The alr3758 gene encodes a potential anti-sigma factor antagonist. RNA-seq data for alr3758 shows that it is essentially off in nitrate-containing medium and upregulated early in response to combined-nitrogen deprivation (Table 2). No RNA-seq reads were present for alr3758 in a *∆hetR* background, indicating that its expression is dependent on HetR. The full-length alr3758-*gfp* upstream promoter fusion was expressed at a very low level in WT and *∆hetR* vegetative cells grown on nitrate (Figure 3, 0 h). After removal of combined nitrogen, the reporter fusion showed increased GFP fluorescence in heterocysts of the WT by 21 hours (Figure 3). This suggests that alr3758 is upregulated in a heterocyst specific manner and may be involved in the inactivation of an anti-sigma factor to allow gene expression of heterocyst-specific genes. There are at least three sigma factors that are upregulated in heterocysts after nitrogen deprivation [30], and alr3758 may play a role in allowing one or more of these sigma factors to access their promoters by sequestering or inactivating an anti-sigma factor in heterocysts.

The asl2028 gene encodes a hypothetical protein that is conserved in only a few species of cyanobacteria. The asl2028 gene is upstream of two genes annotated to encode nitrile hydratases, proteins involved in metabolism of nitriles as a combined nitrogen source, and downstream of a gene for a heme biosynthesis protein. In the WT, the full-length asl2028-*gfp* fusion showed moderate GFP fluorescence in uninduced vegetative cells and the truncated promoter showed no GFP fluorescence (Figure 4). In the *hetR* mutant background, GFP fluorescence was evident but quite dim. At 6 and 12 hours after the removal of combined nitrogen in the WT, there was an increase in GFP fluorescence in vegetative and proheterocyst cells along the entire filament (Figure 4). After nitrogen deprivation in the ∆*hetR* strain, fluorescence remained at the low level observed in the uninduced sample, showing that HetR is required for full expression of this promoter. By 21 hours after the removal of combined nitrogen, the asl2028 promoter was still active in WT vegetative cells, but it was off in mature heterocysts.

The promoter-*gfp* fusion data indicate that asl2028 is a vegetative cell specific gene. Our GFP-reporter data provide useful information on cell-type expression patterns and on qualitative differences in expression levels for different promoters and under different conditions such as nitrogen depletion, but these data are not quantitative. Therefore, the changes in expression observed with the asl2028-*gfp* reporter cannot provide conclusive evidence that asl2028 is regulated by HetR. However, quantitative RNA-seq data (Table 2) show a decrease in asl2028 expression in the *hetR* mutant that is consistent with the *gfp*-reporter results. HetR is expressed in vegetative cells and it has been suggested that it may be involved in the repression of some vegetative cell gene targets [31]. The asl2028 gene is possibly an example of a gene that is at least partially upregulated by HetR in vegetative cells. HetR is conserved in non-heterocystous filamentous cyanobacteria [16] and we have observed that the ∆*hetR* strain grows slightly slower and is more clumpy than wild type cells. Therefore, it is possible that HetR has a role in gene expression in vegetative cells, and asl2028 may be a HetR target that plays a part in that role.

The alr2242 gene encodes a protein in the highly conserved NACHT family of NTPases. Proteins containing this domain include proteins involved in signal transduction, DNA binding, and some kinesin motor proteins [32]. Although there was a strong enrichment of the alr2242 5′ and promoter region in our ChIP-seq data, we could not identify a potential HetR binding site upstream of alr2242 with FiMO. It is possible that HetR binds a yet unidentified site in this promoter, possibly in combination with another protein or proteins. RNA-seq data showed a 2.7-fold induction of alr2242 at 21 h after nitrogen deprivation (Table 2). Although there was no clear change in alr2242 message level in the *hetR* mutant in vegetative cells grown with nitrate (0 h), there was a large decrease in the *hetR* mutant at 6 h after nitrogen deprivation (Table 2).

**Figure 5:**
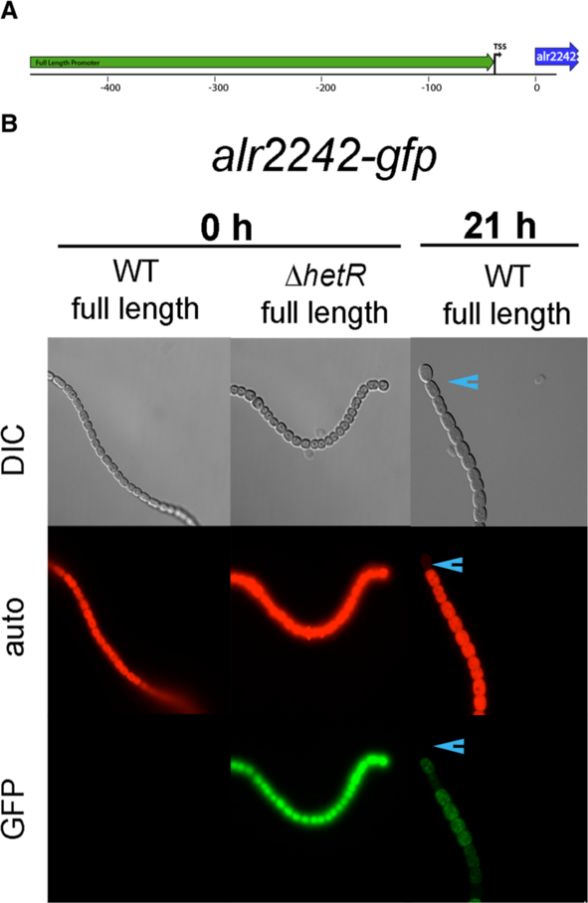
**alr2242 promoter-** ***gfp*** **was upregulated in vegetative cells of the ∆** ***hetR*** **mutant strain. A**. Map of the full-length promoter region upstream of alr2242 that was used to drive GFP reporter expression. Bent arrow labeled TSS marks a transcription start site identified by differential RNA-seq [26]. **B**. The alr2242 full-length promoter fused to *gfp* in WT cells was not expressed prior to nitrogen deprivation (0 h). At 21 hours after nitrogen deprivation, the alr2242 promoter was expressed at a low level in vegetative cells but was off in heterocysts. However, it was strongly expressed in vegetative cells of the ∆*hetR* strain in N + conditions (0 h).

The alr2242 promoter region driving a *gfp* reporter on pAM4662 showed no fluorescence in WT cells grown with nitrate, and weak vegetative cell-specific expression at 21 hours after nitrogen deprivation (Figure 5). No expression was observed in mature heterocysts. However, surprisingly, strong GFP fluorescence was produced in vegetative cells grown with nitrate in the ∆*hetR* mutant background (Figure 5, 0 h). This phenotype was observed for three independent exconjugant strains containing the pAM4662 plasmid. Although there are potential alternative explanations for this result, the simplest conclusion is that HetR suppresses the alr2242 promoter in vegetative cells grown on nitrate-containing medium. In contrast to the promoter-*gfp* fusion data, RNA-seq data showed essentially no difference in alr2242 RPKM values between the WT and the ∆*hetR* strain at 0 h (Table 2). These contrasting results are presumably because the *gfp* reporter data are a measure of promoter activity and the RNA-seq data measure mRNA levels, and suggest that the alr2242 mRNA may be relatively unstable in the *hetR* mutant. Alternatively, the alr2242 promoter-*gfp* mRNA could be an unusually stable transcript, but this effect has never been reported in the large number of studies that have used *gfp*-reporter fusions in *Anabaena*. The alr2242 gene appears to have its own promoter (Figure 5) but potentially could also be expressed as part of an operon with two upstream genes. Further work will be required to understand the regulation of alr2242 and the potential role of HetR in repressing its expression in vegetative cells grown on nitrate.

PatA is a well-studied protein involved in heterocyst development, and previous studies have shown that expression of *patA* increases in a heterocyst-specific manner after nitrogen deprivation [33-36]. *patA-gfp* expression is low in a wild-type background, but in a ∆*patA* strain in N- conditions *patA*-*gfp* is expressed in all cells with the highest expression in heterocysts [34]. The increased expression in the ∆*patA* strain required HetR. This suggests that *patA* is a target of HetR in both vegetative and heterocyst cells and that its accumulation in heterocysts may be due to an increase in HetR levels. A PatA-GFP translational fusion shows that PatA forms FtsZ-like rings around the middle of cells [34]. A ∆*patA* strain only differentiates heterocysts at the ends of filaments in N- media [35], but *patA* overexpression produces aberrant cell morphology and increased heterocyst frequency [34]. Therefore, it has been suggested that PatA is involved in the coupling of cell division and heterocyst differentiation [34].

PatA is expressed in vegetative cells prior to nitrogen deprivation and its expression increases in heterocysts in N- media [34]. Our *in vivo* ChIP data identified a ChIP-seq peak region associated with the *patA* gene, which is consistent with recent bioinformatic analysis that identified a potential HetR binding site upstream of *patA* [10]. RNA-seq analysis of a ∆*hetR* strain showed an 8-fold decrease in *patA* transcription in the absence of HetR (Table 2). It is possible that *patA* and *hetR* regulate one another through a feedback loop both in vegetative cells and during heterocyst development, as both of these DNA-binding proteins appear to affect one another in overexpression and gene knockout experiments [34]. Our ChIP-seq data support the hypothesis that HetR directly regulates *patA* transcription during the response to nitrogen deprivation.

## Conclusions

We employed a combination of ChIP-seq and RNA-seq to examine the HetR regulon at six hours after nitrogen deprivation, when HetR is known to be involved in the activation of some heterocyst-specific genes. The ChIP-seq data produced a set of 26 regions that potentially are bound by HetR protein. Twenty of these regions were found to contain one or more putative HetR binding sites, and analysis of the resulting 59 sites was used to produce a consensus HetR binding site (Figure 1). The genes near the 26 ChIP peak regions include several that have been reported to contain HetR binding sites, including *hetR*, *hetP*, and *patA*, but most of the genes represent new potential HetR targets. Future ChIP-seq studies from different times during heterocyst development may result in identifying additional HetR targets because it is likely that HetR binding to DNA is affected by HetR concentration and influenced by other factors that may be present at different times or under different conditions than those used in this study.

These ChIP-seq data have expanded our understanding of the HetR regulon and identified new promoter regions that are potentially regulated by HetR, including some that do not contain a canonical HetR binding site. Using *gfp* reporter fusions to selected promoter regions potentially regulated by HetR, we found that the asr1469 promoter is transiently upregulated in heterocysts, the alr3758 promoter is upregulated in heterocysts, and the alr2242 promoter is derepressed in nitrate-grown vegetative cells of a *hetR* mutant strain. The *hetR* and *patS* genes are present in some cyanobacterial strains that do not make heterocysts [16], and the potential HetR targets identified in this study may include genes that are regulated by HetR in vegetative cells, which could guide future studies to determine HetR’s role outside of heterocyst development.

## Methods

### Cell growth conditions and nitrogen deprivation

*Anabaena* (*Nostoc*) sp. strain PCC7120 cultures were grown in 100 ml of liquid medium in 250-ml flasks with cotton plugs or in 2 ml of medium in loosely capped tubes as previously described with slight modifications [37]. Briefly, 100-ml or 2-ml liquid cultures were grown to an OD_750_ of 0.05 in BG-11(NH_4_) medium, which lacked sodium nitrate and contained 2.5 mM ammonium chloride and 5 mM MOPS (pH 8.0). For nitrogen deprivation, cultures were spun down at 4,000 × g for 5 minutes and washed three times in BG-11_0_ media by centrifugation and decanting of the supernatant. Cells were then resuspended in 100 ml or 2 ml BG-11_0_ at a final OD_750_ of between 0.02 and 0.05. Cells were grown in liquid BG-11_0_, shaking, with illumination at 100-μmol photons m^−2^ s^−1^.

### Chromatin pull-down

Six hours after nitrogen deprivation, HetR-6xHis cells (*hetR* mutant strain UHM103 carrying pAM4375) were spun down at 4,000 × g for 5 minutes and then resuspended in 5 mL BG-11_0_. Cells were cross linked by the addition of 4.1 mg disuccinimidyl glutarate (DSG) and 0.56 mg ethylene glycol bis(succinimidyl succinate) (EGS) in 500 μL DMSO. Crosslinking occurred at room temperature, rocking, for 20 minutes. After 20 minutes, 135 μL of 37% formaldehyde was added for additional protein to DNA crosslinking and left rocking at room temperature for 15 minutes. To quench the reaction, 125 mM glycine was added for 5 minutes at room temperature.

Cells were then spun down at 4,000 × g for 5 minutes at 4°C and washed twice in 30 mL ice cold PBS (137 mM NaCl, 2 mM KCl, 10 mM Na_2_HPO_4_, 1.8 mM KH_2_PO_4,_ pH 7.4). Washed and fixed pellets were resuspended in 500 μL ice-cold binding/wash buffer (100 mM NaHPO_4_, 600 mM NaCl, 0.02% Tween 20, 1 EDTA Proteinase Inhibitor Tab, Roche Biosciences, in 10 mL total volume) on ice. Protein was extracted by bead beating 2 × 5 minutes with 2 minutes on ice in between. Complete lysis was confirmed by microscopy. Lysed cells were separated from beads via centrifugation and DNA was sheared via sonication on ice, 12 cycles of 20 seconds on, 15 second off at 14% power. Cell debris was pelleted via two cycles of centrifugation at 14,000 × g for 15 minutes at 4°C. Protein concentration was determined by absorbance at 280 nm and normalized to 20 mg/mL for each sample by dilution in cold binding/wash buffer. WT control cells were collected and processed in parallel with the HetR-6xHis cells except that the WT cells were collected at eight hours after nitrogen deprivation because they were being used to control for additional ChIP-seq samples collected at different times.

His-tagged HetR was bound to Dynabeads (Dynabeads His-tag Isolation and Pull-down beads, Invitrogen) following the manufacturer’s protocol at 4°C and eluted in 100 μL elution buffer (100 mM imidazole, 50 mM NaPO_4_, 300 mM NaCl, 0.01% Tween 20). Crosslinks were reversed at 65°C for 18 hours. The bound DNA size distribution was determined on a 1% agarose gel. IP efficiency was measured via western blotting of HetR-6xHis with the Qiagen Penta-His antibody, BSA Free. After crosslinks were reversed, proteins were digested by the addition of 250 μL TE, 4 μL of 20 μg/μL glycogen, and 10 μL of 10 μg/μL proteinase K for 2 hours at 37°C. DNA was column purified with the Promega SV DNA purification kit and resuspended in 30 μL nuclease free water.

### DNA library preparation and sequencing

DNA was prepared for sequencing with the Illumina ChIP-seq Sample prep kit by the Next Generation Sequencing Core at The Scripps Research Institute (La Jolla, CA) following the manufacturer’s protocol. Sequencing was performed on the Illumina HiSeq platform with 4 samples multiplexed on one cell, yielding approximately 40 million 40-bp reads per sample. Sequence reads were demultiplexed based on index sequences and saved as FASTA files for analysis in CLC Genomics Workbench 5.

### Sequence alignment and peak finding

Sequencing reads from the experimental HetR-6xHis sample were randomly assigned to three files and the full ChIP-seq analysis was performed on each sample as a technical replicate. ChIP-seq reads from the three HetR-6xHis samples and the WT sample were aligned to NCBI’s current build of the *Anabaena* genome with CLC Genomics Workbench 5. ChIP peaks were called using a 100 bp window and a false discovery rate of 5% with the WT sample as the control using CLC’s ChIP Analysis pipeline. ChIP peaks were excluded from the final data set if they were not present in at least two of the three technical replicates. All peaks were verified by eye prior to further analysis.

### Promoter-*gfp* reporter fusion construction

Promoter regions for five potential HetR target genes were defined based on genome organization and RNA-seq datasets [25,26], and the region was amplified from the *Anabaena* genome with addition of SacI and SmaI restriction sites using the oligos in Table 3. Truncated versions of four of these promoters, missing the putative HetR binding site, were also amplified with a “truncated” reverse primer (Table 3). PCR fragments were cloned into the SacI and SmaI sites of pAM1956 to yield pAM4653, pAM4654, pAM4658, pAM4659, pAM4660, pAM4662, pAM4695, and pAM4696 (Table 4). Plasmids were then transformed into *E. coli* strain AM1359 for conjugation into *Anabaena* wild type strain AMC1078 and the *∆hetR* strain UHM103. Exconjugants were maintained in liquid BG-11 N + in 2 ml cultures as described above. 2-ml cultures were grown in 24 well plates for nitrogen deprivation. Plasmid constructions were confirmed by DNA sequencing.

**Table 3:** **Oligonucleotides used in this study**

**Oligo Name**	**Sequence**
asr1469FSacI	cagtaggagctcTGAGGAGCGCGAAAGAAAAAAGAG
asr1469RSmaI	cagtaggggcccAAGGCACAAGAGTTTCCATC
asl2028FSmaI	cagtaggggcccCATCTTGCTACAGAATAAAAAAGTTC
asl2028RSacI	cagtaggagctcTGCCGAATTGTGTCGCGG
alr2242FSacI2	gtcagtcccgggCGAGTTGATGAAGTATATCG
alr2242RSmaI4	gtcagtgagctcCTATCAATTATATTTAGTTTTTAAATTAGCGGC
alr3758FSacI	cagtaggagctcTTATTCAAGCTGTTTGGAGTG
alr3758RSmaI	cagtaggggcccAGCTGTTTTTTTCTTTAAATAAATTG
patATruncSacIR	cagtaggagctcTAACTATACGTGTAGTAC
asr1469TruncSacIF	cagtaggagctcTTAAAAAGGTTTTAATTC
asl2028TruncSacIR	cagtaggagctcTTACTGCCACCATCGCGCCCTAG
alr3758TruncSacIF	cagtaggagctcTCAATAGTTCCTGGGCTG

**Table 4:** **Strains and plasmids used in this study**

**Strain**	**Description**	**Source or reference**
AM1359	*E. coli* DH10B carrying pRL623 and pRL443 for biparental conjugations, Tc^r^ Cm^r^ Em^r^ Ap^r^	[12]
AMC1078	*Anabaena* sp. strain PCC7120 Wild Type	Lab strain
UHM103	*Anabaena* PCC7120 *∆hetR* (our strain designation AMC1537)	[28]
**Plasmid**	**Description**	**Source or reference**
pAM505	Shuttle plasmid for expression in *Anabaena,* Km^r^ Nm^r^	[12]
pAM1956	Promoterless gfpmut2 in pAM505, Km^r^ Nm^r^	[12]
pAM4375	pAM505 carrying C-terminal 6xHis tagged HetR, Km^r^ Nm^r^	[11]
pAM4653	pAM1956 carrying the promoter of asr1469 driving GFP, Km^r^ Nm^r^	This work
pAM4654	pAM1956 carrying the truncated promoter of asr1469 driving GFP, Km^r^ Nm^r^	This work
pAM4659	pAM1956 carrying the promoter of asl2028 driving GFP, Km^r^ Nm^r^	This work
pAM4660	pAM1956 carrying the truncated promoter of asl2028 driving GFP, Km^r^ Nm^r^	This work
pAM4662	pAM1956 carrying the promoter of alr2242 driving GFP, Km^r^ Nm^r^	This work
pAM4695	pAM1956 carrying the promoter of alr3758 driving GFP, Km^r^ Nm^r^	This work
pAM4696	pAM1956 carrying the truncated promoter of alr3758 driving GFP, Km^r^ Nm^r^	This work

### Imaging GFP promoter fusions

Promoter-*gfp* fusions of potential HetR targets in WT and *∆hetR* backgrounds were grown in 2 ml BG-11(NH_4_) liquid media in a 24 well plate for 24 hours prior to nitrogen deprivation at an OD_750_ of 0.01-0.03. After nitrogen deprivation was performed as previously described, cells were imaged in the DIC, TRITC (autofluorescence), and GFP channels on an Olympus IX-71 inverted microscope with a 60X objective using Applied Precision’s softWoRx software. Exposure for each channel, GFP, TRITC, and DIC, is the same for each time point of a given strain, and the exposure was the same for all strains except those carrying the promoter of alr2028, where the GFP exposure was 1/5 that of the other strains. Images were false colored red for TRITC and green for GFP in ImageJ 64 [38]. At least 100 heterocysts were scored for determinations of percent heterocysts showing reporter fluorescence.

### Ethics statement

This work did not involve human subjects, human material, or human data and therefore does not require ethical approval.

### Availability of supporting data

The data sets supporting the results of this article are available in the NCBI Gene Expression Omnibus repository, accession GSE51886.

## Additional files

Additional file 1: Table S1. HetR ChIP peaks. Additional file 2: Table S2. Putative HetR binding sites identified with FiMO.

## Abbreviations

ChIP: Chromatin immunoprecipitation or pull-down
DIF: Differentiation related
FDR: False discovery rate
FiMO: Find Individual Motif Occurrences
GO: Gene ontology
Mch: Multiple contiguous heterocysts
TSS: Transcription start site
UTR: Untranslated region
WT: Wild type
