# Supplementary material for: Deep sequencing of HetR-bound DNA reveals novel HetR targets in Anabaena sp. strain PCC7120
Source: BMC Microbiol. 2014 Oct 3;14:255. doi: 10.1186/s12866-014-0255-x (PMC4192349; doi:10.1186/s12866-014-0255-x)
Supplement: Additional file 1: Table S1. — HetR ChIP peaks. [file 12866_2014_255_MOESM1_ESM.pdf]

Additional Table 1. HetR ChIP Peaks.

Region	Length	FDR (%)	# Reads	# Forward reads	# Reverse reads	Normalized difference	Wilcoxon filter p-value	5' gene	3' gene	Overlapping annotations
+(136127..136558)	431	4.755639	54	23	31	0.14814815	0.14100388	all0131 (223)	alr0132 (64)	
+(374646..375090)	444	0.421362	112	69	43	0.23214286	0.01353232	all0326 (145)	all0327	all0327, all0327, Glutamine amidotransferases class-II (Gn-AT)_YafJ-type. YafJ is a glutamine amidotransferase-like protein of unknown function found in prokaryotes, eukaryotes and archaea. YafJ has a conserved structural fold similar to those of other class II...;
+(613436..613919)	483	1.51E-07	385	218	167	0.13246753	0.06926288	alr0520 (881)	patA	patA, patA
+(645374..645809)	435	3.868301	25	18	7	0.44	0.00774085	alr0553	alr0554 (114)	alr0553, alr0553, Integrase core domain; Region: rve; cl01316
+(801575..802006)	431	3.35602	313	145	168	0.07348243	0.27343214	hypF	hupC (999)	hypF, hypF, Hydrogenase maturation factor [Posttranslational modification, protein turnover, chaperones]; Region: HypF; COG0068, yrdC domain; Region: Sua5_yciO_yrdC; cl00205
+(1170772..1171203)	431	3.868301	89	42	47	0.05617978	0.48688744	alr0999	alr1000 (745)	alr0999, alr0999, Transposase DDE domain; Region: Transposase_11; cl09168
+(1729122..1729569)	447	0.133137	239	115	124	0.0376569	0.00023451	nifS (12385)	fdxN	fdxN, all1467, fdxN, all1467
+(1732738..1733693)	955	8.58E-30	1787	880	907	0.01510912	0.24284409	nifS (16001)	fdxN	fdxN, asr1469, alr1470, fdxN, asr1469, alr1470
+(1734763..1735224)	461	0.313089	70	36	34	0.02857143	0.96390246	nifS (18026)	fdxN	fdxN, all1471, all1472, fdxN, all1471, all1472
+(1788499..1788932)	433	3.321759	46	24	22	0.04347826	0.00030131	alr1527	alr1528 (1314)	alr1527, alr1527
+(1890160..1890654)	494	6.02E-58	1333	710	623	0.06526632	5.0436E-11	alr1607 (667)	all1608	all1608, alr1609, all1608, alr1609, Probable transposase; Region: Transposase_2; pfam01385, Transposase and inactivated derivatives [DNA replication, recombination, and repair]; Region: COG2464

+(1890763..1891564)	801	6.9E-57	2654	1279	1375	0.03617182	0.97186731	alr1609	all1610 (610)	alr1609, alr1609, Transposase and inactivated derivatives [DNA replication, recombination, and repair]; Region: COG3464, Transposase; Region: Transposase 12; pfam01610
+(2075925..2076785)	860	7.53E-13	208	98	110	0.05769231	0.00022777	alr1726	alr1727	alr1726, alr1727, alr1726, alr1727, Transposase and inactivated derivatives [DNA replication, recombination, and repair]; Region: COG3415, Integrase core domain; Region: rve; cl01316
+(2216679..2217151)	472	3.868301	9	5	4	0.11111111	0.992	alr1852 (33)	alr1853	alr1853, alr1853, Transposase and inactivated derivatives [DNA replication, recombination, and repair]; Region: COG3415

+(2274728..2275590)	862	1.16E-09	151	72	79	0.04635762	0.00838309	alr1903	cyaB2 (1910)	alr1903, alr1903, FOG: HEAT repeat [Energy production and conversion]; Region: COG1413, Armadillo/beta-catenin-like repeats. An approximately 40 amino acid long tandemly repeated sequence motif first identified in the Drosophila segment polarity gene armadillo; these repeats were also found in the mammalian armadillo homolog beta-catenin...; Region: ARM; cd00020, protein binding surface; other site, Armadillo/beta-catenin-like repeats. An approximately 40 amino acid long tandemly repeated sequence motif first identified in the Drosophila segment polarity gene armadillo; these repeats were also found in the mammalian armadillo homolog beta-catenin...; Region: ARM; cd00020, FOG: HEAT repeat [Energy production and conversion]; Region: COG1413, protein binding surface; other site, Armadillo/beta-catenin-like repeats. An approximately 40 amino acid long tandemly repeated sequence motif first identified in the Drosophila segment polarity gene armadillo; these repeats were also found in [illegible]
+(2427714..2428147)	433	0.006271	276	144	132	0.04347826	0.79166253	all2026 (487)	all2027	all2027, asl2028, all2029, all2027, asl2028, all2029

+(2691579..2692024)	445	0.401938	332	162	170	0.02409639	5.1292E-14	all2239 (199)	alr2240	alr2240, alr2240, Signal receiver domain; originally thought to be unique to bacteria (CheY, OmpR, NtrC, and PhoB), now recently identified in eukaroytes ETR1 Arabidopsis thaliana; this domain receives the signal from the sensor partner in a two-component systems...; Region: REC; cd00156, active site, phosphorylation site, intermolecular recognition site; other site, dimerization interface; other
+(2693489..2693944)	455	0.679439	81	47	34	0.16049383	0.00502315	alr2241 (108)	alr2242	alr2242, alr2242, Predicted NTPase (NACHT family) [Signal transduction mechanisms]; Region: COG5635
+(2706756..2707187)	431	3.868301	52	26	26	0	0.99990556	gvpA (89)	asr2255	asr2255, asr2255, Inositol polyphosphate kinase; Region: IPK; cl12283
+(2820376..2821054)	678	0	2977	1762	1215	0.18374202	0.98777893	alr2338	hetR (583)	alr2338, alr2338, EamA-like transporter family; Region: EamA; cl01037
+(2821271..2822006)	735	0	7395	3700	3695	0.00067613	0.99998352	alr2338 (582)	hetR	hetR, hetR, Peptidase family S48; Region: Peptidase_S48; cl11616
+(2822194..2822924)	730	0	4561	2247	2314	0.01468976	0.00086557	hetR	all2340	hetR, all2340, hetR, all2340, Peptidase family S48; Region: Peptidase_S48; cl11616, aspartate aminotransferase; Provisional; Region: PRK07681, Aspartate aminotransferase family. This family belongs to pyridoxal phosphate (PLP)-dependent aspartate aminotransferase superfamily (fold I). Pyridoxal phosphate combines with an alpha-amino acid to form a compound called a Schiff base or aldimine...; Region: AAT like;
+(3262532..3262973)	441	0.401938	127	56	71	0.11811024	0.25919671	alr2680	all2681 (4507)	alr2680, alr2680, Polyketide synthase modules and related proteins [Secondary metabolites biosynthesis, transport, and catabolism]; Region: COG3321, PKS_DH; Region: PKS_DH; smart00826

+(3287842..3289794)	1952	0	5904	2934	2970	0.00609756	1.1211E-10	alr2697	alr2698	alr2697, alr2698, alr2697, alr2698, Transposase and inactivated derivatives [DNA replication, recombination, and repair]; Region: COG3464, Transposase; Region: Transposase_12; pfam01610
+(3431297..3432038)	741	1.54E-09	349	177	172	0.01432665	7.5789E-13	hetC (99)	hetP (325)	
+(3627499..3627977)	478	1.22E-12	161	72	89	0.10559006	0.79079616	alr2986	alr2987 (2059)	alr2986, alr2986, FOG: HEAT repeat [Energy production and conversion]; Region: COG1413
+(3695599..3696450)	851	1.73E-54	1418	696	722	0.01833568	0.3610782	asr3053	all3054	asr3053, all3054, asr3053, all3054, Uncharacterized protein conserved in bacteria [Function unknown]; Region: COG3330
+(3821175..3821606)	431	3.17516	134	54	80	0.19402985	0.00780868	alr3156	aphA	alr3156, alr3156
+(4383426..4383866)	440	1.288676	114	67	47	0.1754386	0.28731196	avaIR (630)	avaIM	avaIM, avaIM, S-adenosylmethionine-dependent methyltransferases (SAM or AdoMet-MTase), class I; AdoMet-MTases are enzymes that use S-adenosyl-L-methionine (SAM or AdoMet) as a substrate for methyltransfer, creating the product S-adenosyl-L-homocysteine (AdoHcy)...; Region:
+(4539927..4540364)	437	0.702288	57	31	26	0.0877193	0.14153347	alr3757 (11)	alr3758	alr3758, alr3758, Sulphate Transporter and Anti-Sigma factor antagonist) domain of anti-anti-sigma factors, key regulators of anti-sigma factors by phosphorylation; Region: STAS_anti-anti-sigma_factors; cd07043, anti sigma factor interaction [illegible]

+(4740704..4741151)	447	0.008364	199	119	80	0.1959799	0.00219011	alr3926 (1150)	all3927	all3927, all3927, Predicted GTPases (dynamin-related) [General function prediction only]; Region: COG0699, Era (E. coli Ras-like protein)-like. This family includes several distinct subfamilies (TrmE/ThdF, FeoB, YihA (EngG), Era, and EngA/YfgK) that generally show sequence conservation in the region between the Walker A and B motifs (G1 and G3 box motifs)...; Region: Era_like; cd00880, GTP/Mg2+ binding site; other site, Switch II region; other site, G4
+(4857231..4857672)	441	0.426777	120	71	49	0.18333333	0.88257483	alr4031	alr4032	alr4031, alr4032, alr4031, alr4032, ABC-type Fe3+-hydroxamate transport system, periplasmic component [Inorganic ion transport and metabolism]; Region: FepB; COG0614, Transmembrane subunit (TM), of Periplasmic Binding Protein (PBP)-dependent ATP-Binding Cassette (ABC) transporters involved in the uptake of siderophores, heme, vitamin B12, or the divalent cations Mg2+ and Zn2+. PBP-dependent ABC transporters consist...; Region:
+(5064797..5065242)	445	1.19299	53	31	22	0.16981132	0.47840662	asr4228 (209)	alr4229	alr4229, alr4229, Uncharacterized conserved protein [Function unknown]; Region: COG1479, Protein of unknown function DUF262; Region: DUF262; pfam03235
+(5303024..5303480)	456	0.537821	123	48	75	0.2195122	0.74985026	all4423 (998)	all4424	all4424, all4425, all4424, all4425, Predicted glycosyltransferases [General function prediction only]; Region: COG1216
+(5309952..5310383)	431	1.19299	51	13	38	0.49019608	6.9855E-06	hlpA (1327)	all4430	all4430, all4431, all4430, all4431, Predicted glycosyltransferases [General function prediction only]; Region: COG1216

+(5350684..5351627)	943	6.9E-62	395	174	221	0.11898734	0.27629633	all4464 (480)	all4465	all4465, all4465, Transposase and inactivated derivatives [DNA replication, recombination, and repair]; Region: COG0675, Probable transposase; Region: Transposase_2; pfam01385
+(5355615..5356047)	432	3.160056	213	109	104	0.02347418	0.2376336	alr4469	asr4470	alr4469, asr4470, alr4469, asr4470, Glyoxalase/Bleomycin resistance protein/Dioxygenase superfamily; Region: Glyoxalase; cl00411
+(6129044..6129475)	431	4.436927	229	114	115	0.00436681	4.3185E-05	all5131 (34)	all5132 (22)	
