# Supplementary material for: Deep sequencing of HetR-bound DNA reveals novel HetR targets in Anabaena sp. strain PCC7120
Source: BMC Microbiol. 2014 Oct 3;14:255. doi: 10.1186/s12866-014-0255-x (PMC4192349; doi:10.1186/s12866-014-0255-x)
Supplement: Additional file 2: Table S2. — Putative HetR binding sites identified with FiMO. [file 12866_2014_255_MOESM2_ESM.pdf]

Additional Table 2. Putative HetR binding sites identified with FiMO.

ChIP Region	Length	FDR (%)	5' gene	3' gene	Binding site sequence
0136127..0136558	431	4.755638971	all0131	alr0132	GCGGGTAATAACGC
					AAGCGTTATTACCC
					ATGGTTACAAATCA
					ATGGGATTTTAGCA
0613436..0613919	483	1.50628E-07	patA	alr0522	TTGGCTCAAACCCA
					ATGGGTTTGAGCCA
1729122..1729569	447	0.133137459	all1467	alr1468	ATGGGTATTACCAA
					CTGGGTATTACCTT
					ATTGGTAATACCCA
1732738..1733693	955	8.57576E-30	asr1469	alr1470	AAGGCTAAAAACCC
					GTGGGTTTTTAGCC
					TTGGGTCTAACTTA
					TTGGATTTTTACCT
1734763..1735224	461	0.313089483	all1471	all1472	TTGTGATTAACCCT
1788499..1788932	433	3.321759198	alr1527	none close	GTGTGTAAAAACCA
					TTGGTTAATAACAA
2427714..2428147	433	0.006270929	all2026	all2027	CGGGGTAACAATCC
					AAGGATTGTTACCC
2691579..2692024	445	0.401937742	all2239	alr2240	GTGGCTGATGACCT
2820376..2822006	678	0	alr2338	hetR	CCGGGTGCAGCCCA
					AAGGGTTTAACCTT
					TGGGGACTTGACCA
					CGGGGTGTTATCCT
					ATGGGTGTATACCA
					CCGGGTGCAGCCCA
					ACGGGTCAAAATCA
					ATGGCTTCATCCCA
					GTTGGTATACACCC
					TTGGGGAATGCCCT
3431297..3432038	741	1.54167E-09	hetC	hetP	AGGGGTCTAACCCC
					AGGGGTTAGACCCC
					CTGGGTACAATCCT
					TGGGGAAAAACCAC
					ATGGTTGCTTACCA
					GTGGGGAAAAACCA
					GTGGTTTTTCCCCA
					AGTGGTTTTTCCCC
					AAGGGTATAAACAG
					GGGTGTCGTAACCC
					AGGGGATTATAGCC
					CTGGGTTACGACAC
					TAGGATTGTACCCA
					TAGCGTATAACTCA
3627499..3627977	478	1.21738E-12	alr2986	alr2987	ACGTGTAATACCCA
3695599..3696450	851	1.72525E-54	asr3053	all3054	GCGGGTAGATATCC
					TGGGGAATTGACAC
					ATGGTAGCTTACCC
3821175..3821606	431	3.175160382	alr3156	aphA	CTGGTTCTAACTCC
4383426..4383866	440	1.288675926	avaIR	avaIM	TAGGGTTCAAATCT
					ACGGGTATAGCTGA
4539927..4540364	437	0.702287678	alr3757	alr3758	TTGGGTAAACCTGC
					AAGGGTTATTTCCT
4740704..4741151	447	0.008364349	alr3926	all3927	TTGGTTTTTCACCT
5064797..5065242	445	1.192989832	asr4228	alr4229	AAGGGTTAATACTT
5303024..5303480	456	0.537820709	all4424	all4425	AGTGGTATTACGCC
5309952..5310383	431	1.192989832	all4430	all4431	ATGGGTGCAAGCCT
5355615..5356047	432	3.160055785	alr4469	asr4470	AAGGGTTTCAAGCA
					ATGCTTGAAACCCT
					TGGGCTGAAGACCT
